# Honoring the voices of families: An interpretive description of parents’ understandings of, and insights into preventing type 2 diabetes in adolescents

**DOI:** 10.1186/s12887-022-03487-9

**Published:** 2022-07-30

**Authors:** Shelley Spurr, Jill Bally, Nahia Nalwooga

**Affiliations:** College of Nursing, University of Saskatchewan, 104 Clinic Place, Saskatoon, SK S7N 2Z4 Canada

**Keywords:** Prediabetes, Type 2 diabetes, Adolescent, Prevention, Parents, Family, Interpretive description

## Abstract

**Background:**

The incidence of type 2 diabetes (T2D) in adolescents is increasing, affecting the overall health and quality of life of adolescents and their families. Despite the serious health consequences of T2D, few studies have explored the role of parents in the prevention of prediabetes and T2D in adolescents. Thus, the purpose was to better understand parents’ insights into strategies needed for the development of interventions to prevent prediabetes and T2D in their adolescents and families.

**Methods:**

Thorne’s Interpretive Description approach was used to guide this second phase of a two-phase study. Using purposeful sampling, parents (*n* = 12) of adolescents at high risk for developing T2D were interviewed. This study was conducted in accordance with the standards for reporting qualitative research.

**Results:**

Two themes and multiple subthemes emerged: Parents’ Understanding of T2D (High Blood Sugar, Severe Health Impacts, and Managing Your Diet) and It Takes a Village (The Onus is on the Adolescent, Starts at Home with the Parents, We Need More Support, and Getting the Message in Their Face).

**Conclusions:**

These qualitative data were insightful as findings highlighted the parents’ predominant lack of basic knowledge, life skills, and/or resources to prevent prediabetes and T2D in adolescents. The results provided target areas for education and emphasized the importance of using social media as an approach to disseminate important information to adolescents. Parents also identified strategies for prevention interventions for adolescents surrounding prediabetes and T2D that may be effective. Given the increased prevalence of T2D in adolescents, these results are timely and confirm the urgent need for interventions to prevent pediatric prediabetes and T2D. Future research will include the co-design, piloting, and evaluation of feasible family-centered interventions grounded in participants’ experiences and suggestions that are reflective of person-centred goals and needs of adolescents.

## Background

Type 2 Diabetes (T2D) is one of the fastest growing pediatric chronic diseases worldwide [1]. While T2D has typically impacted middle-aged populations and the elderly, more recently it has become increasingly prevalent in children and adolescents [2]. A Canadian national study showed that the minimum incidence of T2D in children < 18 years old was 1.54 per 100,000 per year [1]. Due to the disconcerting incidence, there has been a global recognition of this “new” disease in children [2].

Life-threatening complications of T2D such as diabetic ketoacidosis and hyperosmolar coma often present in adolescents at the time of diagnosis [1, 3]. These accelerated complications are due to the unique aspects of youth-onset T2D such as rapidly progressive β-cell decline [3]. In addition, when T2D is poorly managed, chronic complications such as atherosclerosis, hypertension, heart disease, stroke, neuropathy, retinopathy, and kidney failure are common [1, 4, 5]. Adolescents are especially susceptible to complications due to a combination of early onset, late diagnosis, and poor adherence to treatment [1, 5]. Addressing the increasing incidence of T2D in children and adolescents through preventative interventions is especially critical as complications occur rapidly and are more severe compared to adults [1].

Most research on prediabetes and T2D is quantitative in nature with a focus on incidence, clinical features, treatment, complications, and the factors influencing quality of life [6–8]. However, one recent systematic review explored the use of qualitative methods in diabetes-related research from 1980 to 2010 and found that qualitative methods remain underutilized for understanding the diabetes experience [9]. These authors purport that qualitative methods can provide necessary insight into this chronic disease.

Along with qualitative methods, evidence confirms that family-orientated health promotion is a promising strategy for disease prevention [10]. Barnes et al. [10] found that the family is the best resource for interventions including nutrition, physical and leisure activity, and the utilization of health services. In another quantitative study, Soltero et al. [7] examined a culturally-focused T2D prevention program in Latino families with a child (8–12 years) diagnosed with prediabetes and found a significant decrease in body mass index (BMI) in both parents and children and a reduction in hemoglobin A1c (HbA1c) in parents.

Similarly, a quantitative study by Hingle et al. [6], investigated a family-centered lifestyle behaviour change program with a focus on healthy eating, physical activity, and a supportive home environment to address T2D prevention in children (9–12 years) and their parents. The authors found a reduction in the children’s BMI and improvement in the health of the home environment, especially with regard to nutrition and physical activity [6]. Soltero et al. [7] and Hingle et al. [6] also demonstrated the importance of including family in lifestyle change programs. Despite the increased interest in T2D globally, most intervention studies have focused on lifestyle therapy (physical activity and nutrition) and clinical trials for youths diagnosed with T2D [11, 12]. There remains a paucity of research regarding the prevention of prediabetes and T2D in adolescents and their families.

In addition to the influence that family has on health habits, evidence indicates that family is impacted by individual members' health [8]. For example, a chronic illness suffered by a child can significantly impact the family unit [8]. A quantitative study by Ashleigh et al. [13] examined the relationship between caregiver stress and adolescents' physical and mental health and the protective association between family, community, and health in a sample of Indigenous adolescents diagnosed with T2D in an American state. Results showed that family support and connectedness was positively correlated with the self-reported physical and mental health in these adolescents [13]. Another quantitative study evaluated the perceived health-related quality of life of adolescents diagnosed with prediabetes, T2D, or insulin resistance and their parents [8]. The findings showed that the increased burden of T2D was related to a decline in parents' physical and psychosocial health and family connectedness [8]. Although the positive impact of family has been previously investigated, most studies have been quantitative, focused on adolescents already diagnosed with T2D, and other topics such as overweight/obesity, physical activity, and mental health. Thus, there is a need for more in-depth qualitative research to contextualize the important role parents play in preventing prediabetes and T2D in adolescents and their families. Specifically, while there are qualitative studies about T2D, to our knowledge, no studies have explored perceptions of parents in the prevention of prediabetes and T2D in adolescents and their families. Family inclusion in the prevention of prediabetes and T2D is best explored through use of qualitative methodologies. Qualitative research allows practitioners and researchers to understand people’s experiences, perceptions, and motivations because it provides insight into their beliefs, behaviours, cultures, and lifestyles [9].

The purpose of this two-phase study was to explore prediabetes and T2D in adolescents and their families. Phase one was previously published and used a quantitative approach to examine the prevalence of prediabetes and T2D in adolescents [14]. The purpose of phase two was to explore parents’ understandings and insights into prediabetes and T2D and identify strategies needed for the development of interventions to prevent prediabetes and T2D in their adolescents and families. This research was guided by the following research questions: 1) What are parents’ understandings and experiences of prediabetes and T2D?; and, 2) What do parents feel are appropriate strategies needed to develop interventions at the individual, group, and community levels to prevent prediabetes and T2D in families? The phase two qualitative findings are presented in this article.

## Methods

An interpretive description (ID) approach was used to explore parents’ understandings of prediabetes and T2D and identify appropriate strategies needed to prevent prediabetes and T2D. ID is an inductive qualitative method used to understand the nature and characteristics of phenomenon of interest to health and wellness with the purpose of developing knowledge that is meaningful and easily applied to practice [15]. An underlying assumption of ID is that there are multiple realities that are subjective, socially constructed, and based within a given context [15]. This methodology was suited to this study as the purpose was to understand prediabetes and T2D from the perspectives of parents and to identify suggestions for preventative family-based interventions. Individual interviews were used to gain insight into the parents’ subjective realities.

### Sample

The two-phase research began in January-April, 2018 to identify adolescents at risk for prediabetes and T2D in two urban western Canadian high schools. Recruitment was limited to adolescents (ages 13–19 years) as T2D is predominant in this age range [16]. Adolescents were categorized into high risk using Diabetes Canada [17] criteria including increased blood pressure (hypertension was defined as a diastolic blood pressure (DBP) at or above the 95^th^ percentile for sex, age, and height; those with a systolic or diastolic measure at or greater than the 90^th^ percentile but less than the 95^th^ percentile were considered to have prehypertension) [18], ethnicity (Indigenous, Asian, African, and Arab), overweight/obesity (a BMI greater than 1 standard deviation for their age were considered overweight and those with a BMI greater than 2 standard deviations for their age were considered obese) [19], and HbA1c ≥ 5.5–6.4% (37–46 mmol/mol) were considered high risk for T2D. Results of phase 1 were previously published [14, 20, 21]. In phase 2, all parents of adolescents who presented with ≥ one risk factor for prediabetes and T2D were invited to participate (*n* = 35). Recruitment was halted and considered complete when concurrent analysis revealed that similar responses were being provided, limited new data was being reported, and a thorough understanding of the phenomena was achieved. A purposeful sample of twelve participants were included in this phase of the study.

### Ethical considerations

Ethical approval was received from the University of Saskatchewan Behavioral Ethics Committee, and operational approval was granted from the related school board. To ensure confidentiality, an invitation to participate in the study was sent from the school board office to eligible parents. The letter included information about the purpose and procedures of the study including an explanation of how the interviews would be conducted. All the participants were informed of their rights to withdraw from the study, refuse to answer any questions during the interview, and withdraw any comments. Interested participants contacted the principal investigator by phone or email, as directed by the letter. Written informed consent and assent were obtained from each participant.

### Data collection

Data for this research were collected January-April 2019 and included audiotape-recorded, individual face-to-face, semi-structured interviews with parents, a demographic form, field notes, and memos. The interviews took place in the local high school, in a room that was mutually agreed upon to be comfortable, quiet, and private. All individual interviews lasted 45–60 min. A semi-structured interview guide was used and refined throughout the data collection process based on developing analytical observations [15]. The questions were formulated to provide the participants with opportunities to express their understanding, beliefs, and experiences in relation to T2D and associated interventions. The first author (SS) conducted all interviews. After informed consent was obtained, the facilitator began with questions such as “what does diabetes mean to you?” to elicit the participants’ understandings with as little prompting as possible. Another example of an interview question was; “what do you think your child needs to prevent prediabetes and T2D”. When similar responses were being provided, the facilitator shared some of the ideas and thoughts that had been presented and asked probing questions to allow parents opportunity to expand on the analytical development of the study. For example, the facilitator shared with some of the parents (participants 8–12) an emerging theme that technology should be used to communicate T2D prevention messages to the adolescents. Then, a probing question was used to better understand this idea by asking “what would the message on social media look like”? Concurrently, field notes were documented during and after each interview to record parents’ responses and context as provided by the researcher for use during data analysis [22]. Memos were written by the primary researchers throughout data collection and analysis.

### Data analysis

Data analysis was informed by the analytic techniques found within ID and was completed in a series of steps [15]. Analysis began with multiple readings of the transcribed interviews to allow for the possibility of new discoveries or dimensions with each read. In essence, multiple passes with different agendas were executed to gain a clear understanding of the data. Broad-based categorization was first applied to establish collections of data pieces (initial codes to digitally ‘flag’ that which was deemed meaningful to the research questions) that could be interrogated and determined as thematically related or not [15]. The first readings were completed without any markings or applying artificial categories to the data. Once data was imported, it was then re-read while broad-level codes were generated as they appeared in the data. Larger thematic areas were read through and broken down into sub-themes if they were multi-faceted, with each sub-concept carrying enough weight to warrant its own node, if there was enough evidence to do so. Essentially this final round of coding involved thorough treatment of the data and early coding by taking the initial broad categories and reorganizing, splitting or submerging themes or sub-themes and placing data instances in their most appropriate categories [23]. Once all codes were represented by each category, the interview data was reread again to ensure that the themes represented the opinions of the participants. It is important to note that all quotes extracted for analysis were generated directly from participant language. In other words, in efforts to stay true to the constructed meanings brought forth by participants, their words and their wording only were flagged and coded. The themes were developed with an iterative team process that consisted of a research assistant and two registered nurses all who have extensive experience in pediatrics and/or qualitative research.

### Rigor

The standards for reporting qualitative research checklist (SRQR) was consulted throughout the study to optimize quality [24]. According to Thorne et. al [25], attention to the process and reporting the process is critical to ID, and evaluation of credibility and rigor consists of the several important processes. First, representative credibility was ensured by providing detailed information about the sample, the data collection and analysis procedures, and by triangulation of the data sources to develop a comprehensive understanding (field notes were used to document the context of all data-gathering episodes, memos were written during data collection and analysis). Analytic logic was ensured by transparent decision-making, employing a carefully selected team of researchers which consisted of two nurse researchers and one research assistant who all have experience with interpretive description analysis and keeping an audit trail. It is important to note that the researchers’ consciousness influenced the decisions in the construction of the research and interpretation of the data [22]. However, memos were used to track analytic curiosities throughout the research processes. Additionally, to ensure analytic logic, the data were accurately represented by ensuring that all quotes extracted for analysis were generated directly from participants’ transcripts using direct quotes. Finally, immersion in data was guaranteed with multiple reading, staying open to participant’s subjective perspective, and forming conceptualizations that were grounded in the data and representative of the shared realities to ensure interpretive authority [25].

## Results

This study used a purposeful sample of 12 participants consisting of nine female and three male parents aged 37–57 years were included in this study. Table 1 summarizes the sample demographics including age, gender, and ethnicity of each parent.

**Table 1:** Sample Demographics

Identifier	Age	Gender	Ethnicity
Parent 1	45	Male	Pakistani
Parent 2	37	Female	European
Parent 3	54	Female	Filipino
Parent 4	44	Male	Filipino
Parent 5	Not given	Female	Filipino
Parent 6	46	Female	Filipino
Parent 7	57	Female	Filipino
Parent 8	48	Female	European
Parent 9	53	Female	Russian
Parent 10	51	Female	European
Parent 11	39	Male	Russian
Parent 12	40	Female	European

*Note*. Parent Demographics

The parents shared their understanding and experiences related to T2D, as well as potential strategies that could support the development of interventions to prevent prediabetes and T2D in adolescents. Two main themes and multiple subthemes were identified, described with quotations below and presented in Table 2.

**Table 2:** Themes, Subthemes, and a Sample of Related Parent Quotes

Themes	Sub Themes	Sample of Parent quotes
**Parents’ Understanding of T2D**	High Blood Sugar Severe Health Impacts Managing Your Diet	“Your sugar is high.” “Your body doesn’t regulate your sugar levels.” “He was about 27 years old when he became blind completely from diabetes, and then he ended up with a condition called Shark Foot.” “Do not eat so much of the fast food, just eat a little bit more things that are beneficial to your body like fruits and vegetables and healthier food.”
**It Takes a Village**	The Onus is on the Adolescent Starts at Home with the Parents We Need More Support Getting the Message in Their Face	“Refrain from junk food, make [their] own food, and engage in regular and ongoing physical activity.” “Adolescence is a time where one is old enough to start taking care of [oneself] and [one’s] own health.” “a person still has to want to do it.” “It's up to the parents to monitor and limit what the kids do, like as a far as screen time, and it's also up to the parents to encourage the kids to get exercise and eat properly. So, I just feel so strongly that it starts at home.” “When I see, that it’s 13 g of sugar per this much, well, I don’t know how much sugar I'm supposed to eat in the first place.” “In the class, maybe teachers should give them, like five minutes to stand up and then go jogging, just to boost them.” “We need health care supports to increase and regularize screening, increase staffing (diabetes specialists) in order to shorten wait times, and ensure students are given individual and holistic attention to help catch any particular health problems they might need help with.” “If it’s not coming from home, then we’ve got to get the message in their face, and again I've seen ads on TV that do talk about limiting screen time, and you know they’re just kind of stark messages, but to me they are effective. Because they’re educational. They're trying to reach a mass group.”

### Parental understanding of T2D

According to parents’ responses, T2D involved having poorly regulated *High Blood Sugar* levels that contribute to multiple symptoms and complications resulting in *Severe Health Impacts*. Parents recognized that addressing T2D meant emphasizing behaviour modifications such as *Managing Your Diet* to reduce the health impacts of T2D. However, parents were unable to explain the body’s regulation of blood sugars and had difficulty differentiating between healthy and unhealthy foods.

#### High blood sugar

Most of the parents defined T2D as being associated with elevated blood sugar levels. Explaining what T2D meant, one parent (P7) stated that “your sugar is high” while another parent said (P10) that “your body doesn’t regulate your sugar levels” and one parent (P6) affirmed that “you don’t want that sugar.” However, some of the parents stated that increased blood sugars were related to elevated cholesterol levels (P8), increased intake of sugar especially through sweets (P3), or the body’s inability to produce insulin (P5). One parent (P12) stated, “insulin in the body has something to do with that.” Overall, parents’ responses focused on a person’s consumption and levels of sugar or sweets while only two spoke of the role of insulin in T2D.

#### Severe health impacts

Parents recognized that T2D impacted overall physical and mental health due to its related symptoms and complications that were experienced personally, or by family members and friends. Parents mentioned various symptoms such as thirst, droopy skin, visible ageing (grey skin), leg/foot pain, weight gain/obesity, hypoglycemia (low blood sugar), and sleep disruption. In addition, parents spoke about the observed impacts of diabetes including feeling overwhelmed (P8), blindness (P2), renal failure that necessitates dialysis three times a week (P4), limb amputation (P3,11), “shark foot” (P2), risk of cardiac arrest and heart problems (P4, 12). One parent (P2) shared her father’s experience with T2D and the subsequent complications:

He had juvenile diabetes and found out when he was 14. And then truckers do not take care of themselves - at all. He was about 27 years old when he became blind completely from diabetes, and then he ended up with a condition called Shark Foot? It’s where your arch support gives way, and your foot turns inwards. So, he had that for several years due to diabetes and not taking care of it.

The observed symptoms and complications exemplified the severe health impacts of T2D on their physical health. The parents understood that the occurrence and severity of health impacts were linked to T2D.

#### Managing your diet

Parents understood that with proper management through behaviour modification, people with T2D diabetes can lead normal lives. Although parents talked about physical activity and stress management, the most-discussed behaviour modification was diet management. Parents stated that diets high in sugar and carbohydrates were a risk factor for the development of T2D.

Parents felt that through diet management, people living with T2D could reduce the need for more aggressive therapies such as oral medication and high insulin dosages. However, for T2D prevention, the parents acknowledged that diet management served as an early intervention. One parent (P2) explained what diet management meant to her, “do not eat so much of the fast food, just eat a little bit more things that are beneficial to your body like fruits and vegetables and healthier food.” Prevention through diet management was especially important to parents that had observed symptoms and complications of T2D in family members. *Parents’ understanding of T2D* was limited to physical definitions such as severe impact on physical health and behaviour modifications. Parents identified that modifications to diet were necessary to reduce risk factors of T2D; however, they were unable to state the nutrition content of foods and how they affect well-managed blood sugar levels.

### It takes a village

Based on parents’ responses, prevention of T2D in adolescents involved multiple influences, and they felt that *It Takes a Village* to motivate changes in adolescent health behavior. Parents identified strategies for T2D at the individual, family, and community levels which involved multiple supports such as teachers, medical professionals, community organizations, and peers.

#### The onus is on the adolescent

This subtheme acknowledges that the decision to engage in interventions for T2D prevention ultimately depends on adolescents’ desire to participate in healthy habits including physical activity, healthy eating, and adequate sleep. Speaking about what adolescents can do to reduce the risk of T2D, one parent (P10) suggested that adolescents with T2D should “refrain from junk food, make [their] own food, and engage in regular and ongoing physical activity.” Despite offering suggestions, parents understood that during adolescence, identity is defined outside the context of family and that adolescents begin to make personal decisions. As one parent (P3) explained, “adolescence is a time where one is old enough to start taking care of [oneself] and [one’s] own health.”

Adolescents’ decision-making about their health can be supported through education. Thus, parents thought that diabetes education should be provided at younger ages to support healthy decision-making in adolescence. As one parent (P10) explained, “kids in kindergarten and Grade 1 have the ability to understand, you know, what a healthy food choice is if you teach them.” This subtheme acknowledges that the effectiveness of preventative interventions is dependent on adolescents’ decision to opt in. This same participant elaborated that even with the availability of resources and support to encourage healthy lifestyle changes, “a person still has to want to do it.” Therefore, adolescents need to be motivated through increased awareness of their risk for T2D and education on how their decisions impact personal health.

#### Starts at home with the parents

Adolescents’ adoption of health habits is nurtured by families through encouragement, active role-modeling, and acting as a deterrent for behaviours that increase risk for T2D. As one parent (P10) explained:

It's up to the parents to monitor and limit what the kids do, like as a far as screen time, and it's also up to the parents to encourage the kids to get exercise and eat properly. So, I just feel so strongly that it starts at home.

Parents spoke about encouraging adolescents to engage in health positive behaviour such as physical activity, healthy nutrition, and yearly health monitoring. Parents also mentioned buying home exercise equipment and enrolling adolescents in recreational sports in order to encourage activity. In addition to encouragement, active role-modeling was utilized and involved parents choosing to eat healthier and participate in physical activity to encourage adolescents to follow their example. Thus, active role-modeling often resulted in changes to family habits. One parent (P8) explained her efforts to incorporate healthy foods into family meals:

Every meal has a vegetable with it. Whether it’s like a protein, like a meat? Whether it’s chicken fingers that are breaded, it will have a salad and not the pasta, right? Everything in our house has to have a vegetable.

Parents expressed that encouraging their families to participate in healthy behaviour increased motivation in themselves and adolescents to adopt lifestyle changes.

When encouragement and active role-modeling were unsuccessful, fear tactics were used to deter adolescents from risky behaviours. Parents told adolescents about experienced or observed negative health impacts of T2D to stress the urgency of T2D prevention and the consequences when T2D risk is not taken seriously. A mother (P3) stated:

Sometimes, because I am telling this with the kids sometimes you know what? That’s not, that’s why mom has that because she always like this, like that. So, if you are in the habit of eating like that, you will be getting like this. At age 20 or 25, sometime like that, you will get high blood sugar or diabetes, like your auntie because [your aunt] is always eating sweets.

From childhood, adolescents spend most of their time at home with their parents and, therefore, parents have more opportunities to implement foundational health habits by providing encouragement and serving as role models. However, parents' guidance does not guarantee an adolescent’s engagement in healthy behavior.

#### We need more support

Parents identified a need for education, resources, and involvement of actors such as teachers, medical professionals, community organizations, and peers that can improve access to education, support, and resources to prevent T2D. Parents acknowledged they did not have sufficient knowledge to effectively engage in healthy decision-making to prevent T2D in their adolescent children and families. As one parent (P11) explained, “when I see, that it’s 13 g of sugar per this much, well, I don’t know how much sugar I'm supposed to eat in the first place.” In addition, parents expressed gaps in knowledge related to risk factors, pathophysiology related to prediabetes and T2D, symptoms, complications, healthy eating, and available resources about T2D.

Parents thought that adolescent education about T2D should occur at a younger age since the information the parents learned during their school years has been beneficial in their adulthood. From their experiences, parents suggested that education should include how to read labels, knowledge of the food guide and food groups, and how to identify items with added sugar. Additionally, parents agreed that it would be beneficial to integrate similar information into core curriculum in schools to facilitate adolescents’ healthy decision-making. One parent (P5) stated, “We need to get the Canadian Food Guide implanted in the kids’ heads and which foods are actually healthy and which foods are not. And, help them try to make those right decisions.”

From a young age, adolescents spend much of their time in school. Therefore, parents suggested teachers could play a role in health education, advocacy for healthy meals and snacks, and creation of opportunities for adolescents to engage in healthy activity. As one parent (P7) explained, “in the class, maybe teachers should give them, like five minutes to stand up and then go jogging, just to boost them.”Furthermore, parents thought that teachers should ensure inclusivity to address isolation and bullying which reduces the motivation of at-risk adolescents to engage in physical and social activities. In addition, parents felt that teachers could work in partnership with school counsellors to provide mental and emotional support to adolescents. As one mother (P12) explained:

I'd say access to somebody who the adolescents could talk to at the school. Um, maybe a counsellor or somebody. I know there are counsellors there, but you don’t see them very often in the school. But I know some of the kids there, I think they need it and some of the kids that do bully, maybe if they had that attachment to someone else in the school that they could trust and bond with they would not bully other students.

According to the parents, since teachers spend a lot of time with students, they are in an optimal position to build trusting relationships which increases likelihood that students will listen to T2D education provided by teachers and seek support when experiencing mental health concerns.

Together with teachers, medical professionals work within the healthcare system to address T2D prevention. Parents felt that nurses and physicians play a role in T2D awareness, education, and the completion of annual screening and testing. In addition to nurses and physicians, parents suggested that psychologists provide mental and emotional support and dieticians provide nutritional education. A participant (P2) spoke about the need for input from medical professionals:

We need health care supports to increase and regularize screening, increase staffing (diabetes specialists) in order to shorten wait times, and ensure students are given individual and holistic attention to help catch any particular health problems they might need help with.

Parents also thought that the vigilance of medical professionals was needed to ensure early identification of T2D risk and subsequent assessments. As one parent (P9) explained:

For type 2, I think if they got tested sooner, they can even break it sooner. So, then there won’t be so many people that have it. Then they might make those healthier life choices because they know about it early and took care of it sooner. It is no different than cancer or anything else. If you know about it and you catch it soon enough, you can take care of it.

For parents, the increased availability and involvement of medical professionals was important to ensure early intervention and improve accessibility to education and resources for preventing T2D in adolescents.

Alongside medical professionals, community organizations participate in T2D prevention by implementing free programs and raising awareness of available resources for adolescents and parents. Parents suggested that community programs address education, food preparation, cooking, and physical activity. For example, one parent (P9) suggested a community program that could “get teenagers out at the grocery store and have somebody there to really show them how to shop. They don’t know how to read the ingredients or the weight in grams.” Community programs can be utilized to reinforce education provided in schools and by medical professionals. In addition to community organizations, peer mentorship can provide social and emotional support and motivate adolescents to engage in healthy behaviors. Explaining a peer mentorship program, a parent (P10) stated, “it could be someone their age. Or it could be someone older, and they would be helping them to become more physically active. Or eat healthier.” A participant (P9) said:

A Boys and Girls’ type program where someone aged 27 or 28 would pair up with their child. They could participate in snow shoeing, Ski-Doo-ing, exercises, fishing, and hiking, to name a few activities. But this program would allow parents to join and would require financial backing.

Acknowledging the influence that peers have on adolescents, peer relationships can be utilized to spread awareness and reinforce education about T2D. Overall, parents wanted more free resources, education, and support to address T2D prevention in adolescents. Additionally, parents thought that efforts coming from multiple resources simultaneously would act as reinforcement for healthy habits in adolescents and decrease their risk of developing T2D.

#### Getting the message in their face

The prevention of T2D worldwide requires distributing education in ways that target larger audiences. Therefore, dispensing T2D education through social media would enable easy access to more adolescents. One participant (P10) stated:

If it’s not coming from home, then we’ve got to get the message in their face, and again I've seen ads on TV that do talk about limiting screen time, and you know they’re just kind of stark messages, but to me they are effective. Because they’re educational. They're trying to reach a mass group.

Similarly, another mother (P9) said, “look at the stupid challenges that they have on Facebook and YouTube. They’re slapping people with cheese. How about slapping people with nutrition?” In addition to a wider reach, parents suggested using ads showing worst-case scenarios, which could involve showing people with negative impacts of diabetes to ensure ads are memorable and make adolescents consider behaviour modifications. One parent (P2) said:

You know how they have the stupid pictures on cigarette packages that make it look disgusting? Why can’t diabetes be the same kind of thing, and we post it on social media to show that if you developed diabetes, well this can happen to you? It would be like giving them, I hate to say it, but the worst-case scenario.

Overall, parents thought that ads should capture the potential severity of T2D while being informative. In addition, parents viewed technology and social media as barriers for adolescent engagement in physical activity; however, they acknowledged that adolescents use of social media could be used to widely distribute T2D education and resources. Digital information can be accessed anytime, can be viewed multiple times, and easily shared with peers.

## Discussion

The parents of this study had many ideas about how to prevent diabetes. However, many parents indicated a lack of understanding of what is diabetes.. As a result, families do not have the basic knowledge, life skills, and/or resources to prevent prediabetes and T2D. Specifically, the participants spoke of their *Understandings of T2D* and the meanings they attached to the chronic disease including *High Blood Sugar or* a body’s inability to regulate blood sugar. Only two parents identified that insulin plays a role in blood sugar control. Our study presented new findings as the participants were unable to explain the physiological relationship between high blood sugar, insulin, and T2D. The results are timely and confirm the need for educational interventions along with targeted strategies to prevent prediabetes and T2D in these adolescents and their families Furthermore, these results could help guide the content of education-based interventions that target parents in the prevention of prediabetes and T2D.

Parents also expressed personal and observed concerns about the complications related to T2D including the *Severe Health Impacts*. Our findings showed that parents’ knowledge about potential complications acted as a motivator to engage themselves and their families in healthy behaviors. Parents spoke about buying fruits and vegetables, incorporating healthy foods in family meals, removing sugary drinks from homes, cooking homemade meals, participating in physical activity, enrolling children in sports and taking themselves and adolescents for annual health checkups. Our findings add to the current evidence by confirming parents’ lack in knowledge regarding healthy versus unhealthy sugars and carbohydrates, and the risk factors associated with prediabetes and T2D. Therefore, interventions aimed at T2D prevention in adolescents and their families should address gaps in knowledge related to pathophysiology and nutrition.

A theme emerging from the data was that *It Takes a Village* to prevent prediabetes and T2D. The parents strongly suggested that *The Onus is on the Adolescent.* Most parents expressed that the decision to engage in healthy behaviours ultimately depends on adolescents’ desire and self-motivation to change their unhealthy habits. These results were confirmed by another study that assessed T2D prevention in older adults and showed that motivation to engage in preventative efforts was influenced by awareness of their risk for T2D and increased health education [26].

Most parents acknowledged that health *Starts at Home with Parents* through encouragement, role modeling, and using fear tactics. Parents explained their role in regulation of unhealthy behaviours through limiting screen time and encouraging adolescents to participate in exercise and healthy eating when at home. Previous research has found that T2D prevention programs targeting adolescents were more successful with family involvement [27, 28]. On the contrary, and similar to the parents’ views in this study, the home environment was identified as a barrier to adolescents eating healthy especially when parents’ role-modeled unhealthy eating habits and provide food contradictory to the recommended nutrition requirements for adolescents [6, 27, 28].

In addition to nutrition, parents suggested limiting screen time and role modeling to promote physical activity. When encouragement and role-modeling were unsuccessful, parents in this study reported using fear tactics. Similar to Mulvaney et al. [29], our findings showed that parents use strategies such as sharing stories about family members with serious T2D complications to induce fear in the hopes of dissuading adolescents from unhealthy habits. However, the lasting effect of fear tactics remain unclear. Although most parents were eager to engage in healthy habits, there was a gap in knowledge about how to attain this goal. Our unique findings identified that parents’ education should address specific gaps in knowledge related to healthy food preparation, reading nutrition labels, and healthy sugars, fats, and carbohydrates. Folling et al. [26] showed that increased T2D education correlated with more involvement in healthier habits and increased knowledge-seeking behavior related to T2D prevention. Our results reiterate the importance of targeting parents and specific education topics in future research and programs to prevent prediabetes and T2D.

The parents in this study also identified the important influence of supports including teachers, medical professionals, community organizations, and peers. Our findings confirm previous research that has shown the most suitable environment to implement preventative programs for adolescents is schools [28, 30]. In this setting, teachers have opportunities to provide health education and schools model healthy behaviours [27]. In addition, with limited availability of school counsellors, teachers support adolescent emotional and mental health by addressing issues such as bullying which contribute to unhealthy eating habits and a lack of motivation to engage in physical activity, increasing the risk for T2D [31]. However, our findings add to the current evidence by demonstrating that teachers play a role in advocacy for healthy foods and snacks in school cafeteria and vending machines.

In addition to schools, our data support previous research that identified the vital role of healthcare professionals, community organizations, family, and peers in diabetes education, supportive care (physical, mental and emotional), and ensuring accessibility of resources [30, 32–34]. Previous research has shown that community organizations connect at-risk populations to interventions by improving access to physical activity and programs to prevent T2D [35]. However, our findings expand upon the role of community organizations to include improving access through advocacy and offering free prediabetes and T2D prevention programs to adolescents and their families. In addition, our results identified specific roles health professionals have in implementation of T2D prevention programs such as yearly screening/testing, counselling, and cooking and nutrition classes.

The parents in this study underscored the need for social media to make education, support, and resources more accessible. Elnaggar et al. [36] and Petrovski et al. [37] also showed that social media can be utilized in T1D and T2D self-management. However, our unique findings show parents believe that platforms such as Instagram, Facebook, and YouTube could be employed in T2D prevention to raise awareness and improve education since most adolescents interact with social media daily. Folling et al. [26] found that fear of the negative impacts of T2D is a motivator for behaviour change in older adults. However, Mulvaney et al. [29] showed the contrary in adolescents because they tend to lack consideration of the long-term consequences of their actions believing they can implement health changes later. In addition, our parents suggested that ads targeting adolescents should illustrate the extreme chronic complications of T2D to demonstrate to adolescents the immediate and lasting effects of their health behaviours. Overall, the parents of this study shared insightful suggestions for improving both the adolescents’ and parents’ knowledge and strategies to prevent prediabetes and T2D. Future research should examine the integration and effectiveness of social media in preventing T2D, and explore how parents, teachers, health care professionals, and community organizations identified in this study can collaborate towards prevention of T2D in adolescents.

## Strengths and limitations of the study

The findings of this interpretive description study have important implications for addressing prediabetes and T2D prevention in adolescents and families, and were based on rigorous research processes; however, there are existing limitations. All the parents had adolescents that participated in a previous screening study and were identified as having at least one risk factor for prediabetes and T2D (BMI, HbA1C, BP and ethnicity). Therefore, purposive sampling may have led to biased findings as parents were aware of their adolescents’ risk and may have already initiated some preventative interventions. Nevertheless, parents are major stakeholders in their adolescents' health and, thus, it was crucial to explore their T2D knowledge and efforts to incorporate healthier lifestyles in families prior to engagement in diabetes prevention interventions. Also, adolescents were recruited from two urban Canadian schools in one city. Thus, the findings might not be representative of adolescents and parents in rural areas nor internationally. The use of purposive sampling does not imply the transferability of findings, but it does provide rich data from participants that have experienced the phenomenon of interest [15, 22]. Furthermore, in qualitative research, sample adequacy is indicated when there is a good understanding of the topic of study, which was achieved with a sample size of 12 parents [22]. The sample included mostly mothers, which may limit understanding of the fathers’ perspectives and experiences. However, the involvement of several male participants in this study contributes to addressing the gender bias in nursing research. All interviews were conducted by the first author which may be seen as a strength and limitation. First, there was consistency applied to data collection by having one experienced interviewer; second, it may be that potential biases were repeated amongst all interviews. However, all authors met consistently throughout the data collection phase of the research to discuss the data collected and ongoing analysis, ensuring representative credibility, analytic logic, and immersion [15].

## Conclusions

The data herein showed that the parents did not have the knowledge, life-skills, and resources to prevent prediabetes and T2D in their adolescents and families. The parents identified multiple strategies that could be used in the prevention of prediabetes and T2D including: Targeting parents with specific education topics (pathophysiology, nutrition, risk factors); supporting teachers who can advocate for healthy foods and snacks in school cafeteria and vending machines; improving access to community programs (physical activity and food preparation classes); increased availability and involvement of medical professionals (screening/testing, counselling, and cooking and nutrition classes); and, using social media to make education, support, and resources more accessible. To our knowledge, this is the first study that engaged parents in formative research designed to explore their understandings and strategies to prevent prediabetes and T2D. Given the increased prevalence of prediabetes and T2D, the results are timely and confirm the need for educational interventions along with targeted strategies to prevent prediabetes and T2D in these adolescents and their families. These new and unique findings will be used as the foundation for our future research which will include the co-design, piloting, and evaluation of feasible family-centered interventions grounded in participants’ experiences and suggestions that are reflective of person-centred goals and needs of adolescents and their families.

## Data Availability

The datasets generated and/or analyzed during the study are not publicly available due to confidentiality but are available from the corresponding author on reasonable request.
